# Quantification of Plasma Kynurenine Metabolites Following One Bout of Sprint Interval Exercise

**DOI:** 10.1177/1178646920978241

**Published:** 2020-12-10

**Authors:** Ada Trepci, Sophie Imbeault, Victoria L Wyckelsma, Håkan Westerblad, Sigurd Hermansson, Daniel C Andersson, Fredrik Piehl, Tomas Venckunas, Marius Brazaitis, Sigitas Kamandulis, Lena Brundin, Sophie Erhardt, Lilly Schwieler

**Affiliations:** 1 Department of Physiology and Pharmacology, Karolinska Institutet, Stockholm, Sweden; 2 Institute of Sport Science and Innovations, Lithuanian Sports University, Kaunas, Lithuania; 3 Waters Sverige AB, Stockholm, Sweden; 4 Cardiology Unit, Heart, Vascular and Neurology Theme; Karolinska University Hospital, Stockholm, Sweden; 5 Neuroimmunology Unit, Department of Clinical Neuroscience, Center for Molecular Medicine, Karolinska Institutet, Karolinska University Hospital, Stockholm, Sweden; 6 Division of Neurology, Karolinska University Hospital, Stockholm, Sweden; 7 Center for Neurodegenerative Science, Van Andel Research Institute, Grand Rapids, MI, USA; 8 Department of Clinical Sciences Lund, Faculty of Medicine, Lund University, Psychiatry, Lund, Sweden

**Keywords:** Kynurenine, kynurenic acid, LC/MS/MS, tryptophan-kynurenine pathway, sprint interval exercise

## Abstract

The kynurenine pathway of tryptophan degradation produces several neuroactive metabolites suggested to be involved in a wide variety of diseases and disorders, however, technical challenges in reliably detecting these metabolites hampers cross-comparisons. The main objective of this study was to develop an accurate, robust and precise bioanalytical method for simultaneous quantification of ten plasma kynurenine metabolites. As a secondary aim, we applied this method on blood samples taken from healthy subjects conducting 1 session of sprint interval exercise (SIE). It is well accepted that physical exercise is associated with health benefits and reduces risks of psychiatric illness, diabetes, cancer and cardiovascular disease, but also influences the peripheral and central concentrations of kynurenines. In line with this, we found that in healthy old adults (*n* = 10; mean age 64 years), levels of kynurenine increased 1 hour (*P* = .03) after SIE, while kynurenic acid (KYNA) concentrations were elevated after 24 hours (*P* = .02). In contrast, no significant changes after exercise were seen in young adults (*n* = 10; mean age 24 years). In conclusion, the described method performs well in reliably detecting all the analyzed metabolites in plasma samples. Furthermore, we also detected an age-dependent effect on the degree by which a single intense training session affects kynurenine metabolite levels.

## Introduction

**Figure 1.**
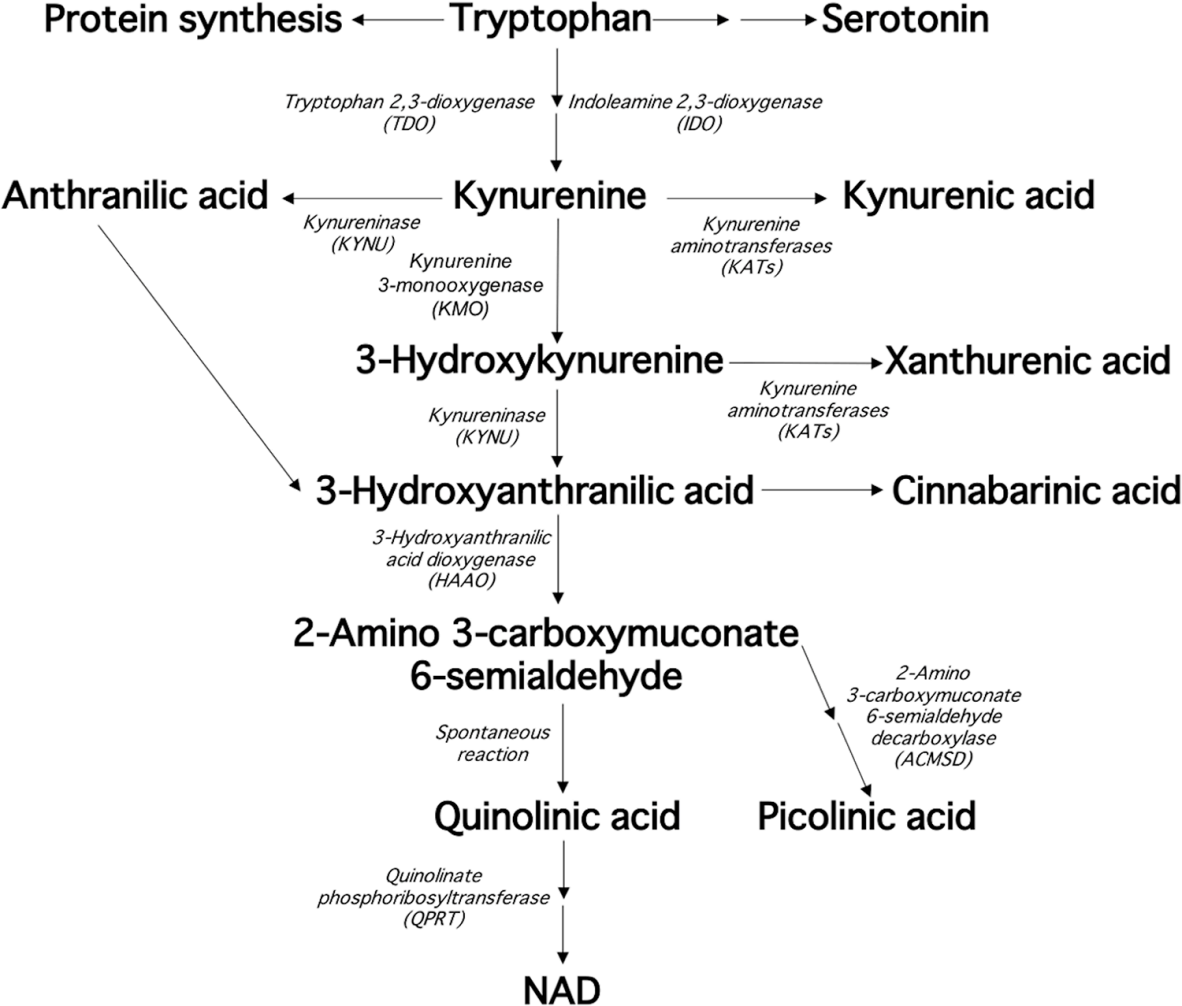
The kynurenine pathway of tryptophan degradation.

The kynurenine pathway is the main route of tryptophan degradation and produces various metabolites, such as kynurenine, kynurenic acid (KYNA), 3-hydroxykynurenine (3HK), xanthurenic acid (XA), 3-hydroxyanthranilic acid (HANA), quinolinic acid (QUIN), picolinic acid (PIC), nicotinic acid (NIC), and nicotinamide (NAA) (Figure 1). The pathway is tightly regulated by enzymes and several of its metabolites are neuroactive with tissue- and cell-specific effects.^1^ Activation of the kynurenine pathway is implicated in a variety of disorders and diseases, including tick-borne encephalitis (TBE),^2^ human immunodeficiency virus infection (HIV),^2,3^ malaria,^4,5^ system lupus erythematosus (SLE),^6,7^ multiple sclerosis (MS),^8,9^ cardiovascular disorder,^10^ and diabetes.^11-13^ Furthermore, activation of the kynurenine pathway is present in psychiatric disorders such as schizophrenia,^14,15^ major depression,^16^ bipolar disorder,^17,18^ and suicidality.^19,20^ Previous studies have demonstrated that both immune activation and polymorphisms in genes for specific kynurenine pathway enzymes can alter the concentration of metabolites.^16,18,20,21^ In addition, physical activity has also been shown to influence the concentration of kynurenine pathway metabolites.^22,23^

Numerous studies have analyzed the content of kynurenine metabolites in plasma and cerebrospinal fluid (CSF) during health and disease as well as following different kinds of exercise. Most of these studies have analyzed only a few metabolites since a robust method for quantitative and simultaneous detection of several metabolites, including stability tests for all metabolites measured, has been missing.^24-32^ We have recently developed a novel, robust, and highly sensitive ultrahigh-performance liquid chromatography tandem mass spectrometry (UPLC-MS/MS) method for simultaneous quantification of ten kynurenine metabolites in the CSF.^25^ In the present study we have further developed this method for quantification of these metabolites in human plasma and analyzed the effect of 1 sprint interval exercise (SIE) on plasma concentration of kynurenine metabolites in young and old male volunteers. SIE is not regarded as traditional endurance training, rather it is characterized by short repeated bouts of high-intensity exercise interspersed with periods of recovery.^26,27^ Previous studies have shown that SIE is a time effective method for improving metabolic health, boosting aerobic capacity and reducing the risk of cardiometabolic disease, such as type 2 diabetes^28,29^ but its effects on kynurenine metabolites are not known. The aims of the present study are first to validate our newly developed UPLC-MS/MS protocol for plasma samples, second to analyze the stability of the kynurenine metabolites in plasma and third to apply the protocol on a cohort of young and old healthy subjects and investigate if 1 SIE session affects the concentration of plasma kynurenine metabolites.

## Material and Methods

### UPLC-MS/MS method validation

#### Chemicals

Normal standards: tryptophan, L-kynurenine, pyridine-2,3-dicarboxylic acid (Quinolinic acid, QUIN), kynurenic acid (KYNA), picolinic acid (PIC), nicotinamide (NAA), nicotinic acid (Niacin, NA), xanthurenic acid (XA), 3-hydroxykynurenine (3-HK), 3-hydroxyanthranilic acid (3-HANA)—were purchased from Sigma-Aldrich (MO, USA). The Internal standards (IS): tryptophan-*d*_3_, L-kynurenine-*d*_4_, QUIN-*d*_3_, [^13^C_6_] NAA, [^13^C_6_]NA, XA-*d*_4_, 3-HK-*d*_3_, 3HA-*d*_3_, were purchased from Toronto Research Chemicals Canada (Toronto Canada). KYNA-*d*_5_ and PIC-*d*_4_ were obtained from C/D/N Isotopes Inc. (Quebec, Canada). Solutions for the mobile phases: water, methanol and formic acid 99% were all LC-MS grade from Chromasolve, Honeywell, VWR International AB, Stockholm (Sweden).

Solutions for plasma preparations: ammonia (32%) was purchased from VWR and zinc sulphate was purchased from Sigma-Aldrich (MO, USA).

#### Standard solutions

Ten stock solutions of all unlabeled standards (tryptophan, kynurenine, QUIN, KYNA, PIC, NAA, NIC, XA, 3-HK and 3-HANA), were prepared in water for HPLC, LC-MS grade and stored at −20°C. Calibrators were generated mixing all compounds in a final solution of 8.3 µM for kynurenine, QUIN, KYNA, PIC, NAA, NIC, XA, 3-HK and 3-HANA and ten times higher for tryptophan, 83 µM. The calibrator mix was then aliquoted in volumes of 200 µl and stored at −80°C. Before each experiment, calibrator mix was thawed and serially diluted in water for HPLC, LC-MS grade before each experiment. The IS stock solutions of all compounds were prepared in water with a final concentration of 4.1 µM (tryptophan-*d*_3_, 41 µM) and stored at −80°C in aliquots of 350 µl.

#### Analysis with UPLC-MS/MS

The UPLC-MS/MS system used was a Xevo TQ-XS triple quadrupole mass spectrometer (Waters, Manchester, UK) equipped with a Z-spray electrospray interface and a Waters Acquity UPLC I-Class FTN system (Waters, Milford, MA). The MS was operated in electrospray positive multiple reaction monitoring (MRM) mode. The conditions were set as follow for the interface: source temperature of 150°C; desolvation gas flow rate 1000 L/h; cone gas flow rate 150 L/h; capillary voltage of 3.0 kV; desolvation temperature 650 C; and detector gain 1. The UPLC condition was as follows: column, Acquity HSS T3 1.8 μm with dimensions 2.1 × 150 mm, (Waters, part number: 186003540) column temperature 50°C; guard column (Waters, Vanguard HSS T3 1.8 μm 2.1 × 50 mm column, part number: 186003976) was installed to retain contaminants from the mobile phase. The mobile phase A was 0.6% formic acid in water (UPLC grade) and the mobile phase B was 0.6% formic acid in methanol (UPLC grade). The flow rate was 0.3 ml/min and the run time for each sample was 13.0 minutes. The autosampler was set at 4°C. Data processing was performed using MassLynx 4.1 software. The software was used for calculating the dwell times for the MRM channels, giving a desired number of 15-20 data points across the chromatographic peak.

The m/z for the MRM transitions of each individual analyte, along with optimal cone voltages and collision energies were determined by manual tuning using the instrument’s built-in fluidics system (MassLynx 4.1 software). A 10 L/minute flow of 100 ng/mL tuning solution was introduced to the mass spectrometer in combination with an LC flow of 0.2 mL/minute and a composition of 20/80 mobile phase A/mobile phase B. The MRM transition providing the highest sensitivity was chosen as quantification trace for all compounds, except for tryptophan and kynurenine where the C13 isotopes were selected to reduce overall signal intensity.

#### Sample preparation

30 μl of human plasma sample, calibrator sample or Quality Control sample was mixed with 30 μl of IS 0.5 μM in 10% Ammonium hydroxide (UPLC grade) solution during 15 seconds and then 60 μl of 200 nM ZnSO_4_ (+5°C) was added and mixed for 15 seconds before 30 μl of Methanol (+5°C) (UPLC grade) was added and mixed for 15 seconds. The mixture was then centrifuged for 10 minutes at 2841 × g at room temperature. Thirty microliters of the supernatant was mixed with 30 μl of formic acid 5% in LC-MS Certified Clear Glass 12 × 32 mm vials (Waters, product no. 186005662CV) before transfer to an autosampler (set to 5°C) that injected 1.5 μl into the UPLC-MS/MS system.

#### Method validation

The method’s selectivity, linearity, accuracy, precision, and matrix effects were validated according to the guidelines from the European Medicines Agency (EMA) and US Department of Health and Human Services Food and Drug Administration (FDA). For chromatographic assays, recommended accuracy and precision variations are ±15% (LLOQ: ±20%) of nominal concentrations. Spiked plasma in 2 different concentrations (low: tryptophan 9 µM; kynurenine 3 µM; KYNA 50 nM; QUIN 300 nM; PIC 70 nM; NAA 150 nM; NA 2 nM; 3-HANA 40 nM; XA 15 nM; 3HK 50 nM) (high: tryptophan 40 µM; kynurenine 1.5 µM; KYNA 500 nM; QUIN 3000 nM; PIC 700 nM; NAA 1500 nM; NA 20 nM; 3-HANA 400 nM; XA 150 nM; 3HK 500 nM) was used in order to test the accuracy and precision of the assay. Accuracy is presented as percentage recovery (100× (measured C_spiked_-C_non-spiked_)/C_spiked)_) and the assay precision is presented as percent relative standard deviation (STDEV (Data Range) / AVERAGE (Data Range)) × 100) and was calculated from repeated measurements within the same experiment (intra-assay, *n* = 6, during 20 hours with samples stored in sample manager at 5°C) or from three different experiments running over two days (inter-assay). The matrix effect was calculated by comparing peak area of each metabolites IS in *pooled plasma* (*n* = 6) in relation to the peak area of pure water sample prepared exactly as plasma (*n* = 6), as follows (peak area (plasma)/peak area (water)−1) × 100%. The matrix effect should be reproducible and consistent.

We investigated the selectivity for all 10 metabolites by comparing chromatograms of extracted blank plasma obtained from 6 different human samples spiked with a mix of all 10 metabolites and IS to ensure that it was free of interference at the retention time of the compounds. The linearity was tested with a calibrator mix diluted in water in concentrations ranging between 0.006 to 8.3 µM (for tryptophan the concentrations were 0.06 to 83 µM). Each calibrator concentration and human plasma sample were analyzed in duplicates. The standard curve was calculated by 1/X weighted least squares linear regression of standard curve calibrator concentrations and the peak area ratios of analyte to IS. A signal-to-noise ratio of 3 and signal-to-noise ratio of ten was used for estimating limit of detection (LOD) and limit of quantification (LOQ) respectively.

#### Stability tests

We investigated the stability of freeze-thaw cycles and the storage at room temperature for different lengths of time. The freeze-thaw stability was evaluated using plasma samples from 6 different subjects stored at −80°C. The samples were thawed to room temperature for 4 cycles with 24 hours in between. Samples were stored at room temperature for up to 4 hours after first thawing (covering a normal time period for laboratory handling during sample analysis).

The stability before and after the first freezing cycle was tested in plasma samples from 4 individual subjects. We analyzed those 4 plasma samples directly after blood withdrawal, and after 2 hours at room temperature, then again after 1 freezing cycle (covering normal handling time and practices for clinical blood samples). Percent stability for each analyte is given as the mean percent stability of all 4 individuals plasma ± SD at the given hour.

#### Stability cohort

Plasma from six subjects enrolled at the MS outpatient clinic at the Karolinska University Hospital between December 2017 and March 2019 was analyzed following storage in −80^o^C (August 2019) and used for testing the stability following repeated freezing- and thawing-cycles as well as following storage at room temperature for up to 4 hours. Plasma from the 4 healthy controls enrolled in 2019 within the Karolinska Schizophrenia Project was used for investigating the impact of the first freezing and thawing cycle, that is, that the plasma was analyzed within 30 minutes after withdrawal and before putting the samples into the freezer. The next day, following 24 hours at either −80^o^C or at room temperature, the same samples were analyzed again. Patients and controls should meet the following criteria: 1. Being 18-55 years of age. 2. No known major somatic or psychiatric diagnoses besides MS for the patient group. 3. No psychiatric or psychotropic medication, including glucocorticoids, within 90 days of sampling.

#### Sprint interval exercise cohort

##### Subjects and exercise protocol

Twenty healthy volunteer males participated in the study. None of the participants were part of any structured sport activity but were recreationally active. Subjects were divided in 2 subgroups, young (24 ± 3.2 years old, mean ± SD) and old (64.3 ± 5.7 years old, mean ± SD). All the participants gave written informed consent before participation. The protocol was approved by the local ethics committee and was conducted following the Declaration of Helsinki.

The exercise bout employed in this study consisted of 1 SIE session. The SIE session started with a warming up session of 8 minutes cycling at a power (W) equal to the individual’s body mass (kg), followed by 6 repetitions of a 30s all-out cycling bout at 0.7 Nm/kg body weight with 4 minutes passive recovery between sprints.^30^

Venous blood samples (5 ml) were collected from the antecubital vein from all subjects before SIE (baseline), as well as 1 hour and 24 hours after SIE. Blood samples were collected in EDTA tubes and centrifuged at 1438 × *g* for 10 minutes. The supernatant of plasma was collected and stored at −80 C until analysis.

The subjects were instructed to maintain their regular diet and not to eat for at least 2 hours before the SIE session.

##### Statistical analyses

All statistical analyses were performed using GraphPad Prism 8 for Mac (GraphPad, La Jolla, Ca, USA). Data are expressed as mean ± SD. Data from the SIE study was analyzed using Mann-Whitney U tests to compare the metabolites at baseline (young vs old). Kruskal-Wallis test was used to analyze the effects of time after SIE on metabolite levels within age groups followed by post hoc Dunn’s test with baseline serving as the control condition. Data are presented as mean ± standard deviation (SD) and the significance of all tests was set at *P* < .05.

## Results

### UPLC-MS/MS method validation

The objective of this study was to validate the selectivity, specificity, sensitivity, linearity, precision, accuracy, and matrix effect of a new LC-MS/MS method for measuring tryptophan and kynurenine metabolites in plasma by following the guidelines for bioanalytical method validation from the US FDA (https://www.fda.gov/files/drugs/published/Bioanalytical-Method-Validation-Guidance-for-Industry.pdf and the EMA (https://www.ema.europa.eu/en/documents/scientific-guideline/guideline-bioanalytical-method-validation_en.pdf).

#### Sensitivity, selectivity and specificity of the UPLC-MS/MS method

Each analyte and IS was infused into the mass spectrometer and tuned for its molecular transitions (see Table 1 for precursor/product transitions for all compounds and IS). The MRM transition providing the highest sensitivity was chosen as quantification trace for all compounds, except for tryptophan and kynurenine where the C13 isotopes were selected to reduce the overall signal intensity. This provided better linearity of the response over the calibration range, since the concentration of tryptophan and kynurenine in biological samples are significantly higher (µM range) than for the other analytes in this method (nM range).

**Table 1.** Transitions and mass spectrometry parameters for all compounds and their internal standards.

Compound/internal standards	Precursor ion mass	Product ion mass	Cone voltage (V)	Collision energy (eV)
Tryptophan	206.1	118	20	24
		146**	20	16
Kynurenine	209.1	94	20	12
		146	20	18
KYNA	190.1	116	30	28
		144	30	17
QUIN	168.1	78	20	18
		124	20	10
PIC	123.9	78*	30	16
		96	30	16
NAA	123	78	25	16
		80*	25	16
3HK	225.2	110.1	14	16
		162.1	14	16
NIC	123.9	80*	25	16
		96	25	16
XA	206.1	160.0	25	16
		132.0	25	30
3HANA	154	108*	20	18
		136	20	10
Tryptophan-*d_3_*	208.1	118.8	40	26
Kynurenine-*d_4_*	213.2	94	30	15
KYNA-*d_5_*	195	121	28	26
QUIN-*d_3_*	171	81	20	18
PIC-*d_4_*	128	82	4	17
NAA [^13^C_6_]	129.1	101	20	16
3HK-*d_3_*	228.2	163	14	16
NIC [^13^C_6_]	130.1	85.2	32	20
XA-*d_4_*	210.1	192	25	10
3HANA-*d_3_*	157	83	24	24

Abbreviations: KYNA, kynurenic acid; QUIN, quinolinic acid; PIC, picolinic acid; NAA, nicotinamide; 3HK, 3-hydroxykynurenine; NIC, nicotinic acid; XA, xanthurenic acid; 3HANA, 3-hydroxyanthranilic acid.

* quantifying ion.

** MS transition using C13 isotopes and product ion with low response were selected to reduce the overall signal intensity.

The five-point standard curves of all ten metabolites tested show strong linearity within the concentration ranges tested (0.006-8.3 µM; tryptophan: 0.06-83 μM), using stable isotope-labeled IS. The correlation coefficient R^2^ of the regression equations for tryptophan, QUIN, KYNA, PIC, NIC, XA, 3-HK and 3-HANA were 0.999, kynurenine and NAA 0.996, and PIC 0.995 (see Table 2). The lowest analyte concentration that could be measured with acceptable accuracy and precision (ie, LLOQ, S/N ratios >10, see Table 1) was in the range that covered stable detection of all metabolites in plasma with an exception for NIC that was detected in fewer than 30% of all samples tested (*n* = 60) and therefore excluded from further analyses.

**Table 2.** Intra-assay (6 repeated analyses within 1 experiment during 20 h with samples stored in sample manager) and inter-assay validation.

Compound	Linearity (R^2^)	LOD-LLOQ	% Matrix effect	Intra-assay (*n* = 6 during 20 h in +4*C)				Inter-assay (*n* = 3)			
				Accuracy (% of target)		Precision (RSD%)		Accuracy (% of target)		Precision (RSD%)	
Tryptophan	0.999	6-6 nM	−38.6	108.2	100.4	1.2	0.9	102.6	85.6	6	6.4
Kynurenine	0.996	6-6 nM	−23.3	108.5	113.1	0.7	2.3	114.8	105.2	2.8	13.6
KYNA	0.999	6-6 nM	−36.7	90.7	91.8	1.8	0.6	87.9	98.0	10.8	6.9
3-HK	0.999	6-10 nM	−54.5	96.3	87.0	1.9	1.2	87.3	85.2	9.5	5.0
QUIN	0.999	6-10 nM	−30	87.3	93.9	2.7	2.6	114.7	103.2	1.9	10.6
PIC	0.995	10-10 nM	−50.5	90.2	99.8	2.4	0.9	86.9	96.0	6.5	7.6
NAA	0.996	6-10 nM	−27.4	104.4	88.1	3.2	2.4	102.5	89.7	1.8	4.5
3-HANA	0.999	6-10 nM	3.2	110.4	107.3	4.4	1.8	112.9	79.2	5.1	14.4
NIC	0.999	10-10 nM	−26.3	104.8	95.9	1.4	1.6	112.5	94.4	9.3	4.5
XA	0.999	6-10 nM	−18.5	100.6	100	5.2	1.4	86.3	105.6	3.7	6.5

Abbreviations: KYNA, kynurenic acid; QUIN, quinolinic acid; PIC, picolinic acid; NAA, nicotinamide; 3HK, 3-hydroxykynurenine; NIC, nicotinic acid; XA, xanthurenic acid; 3HANA, 3-hydroxyanthranilic acid, LOD, limit of detection (S/N ratio of three); LLOQ, lower limit of quantification (S/N ratio of ten); RSD: relative strandard deviation.

**Figure 2.**
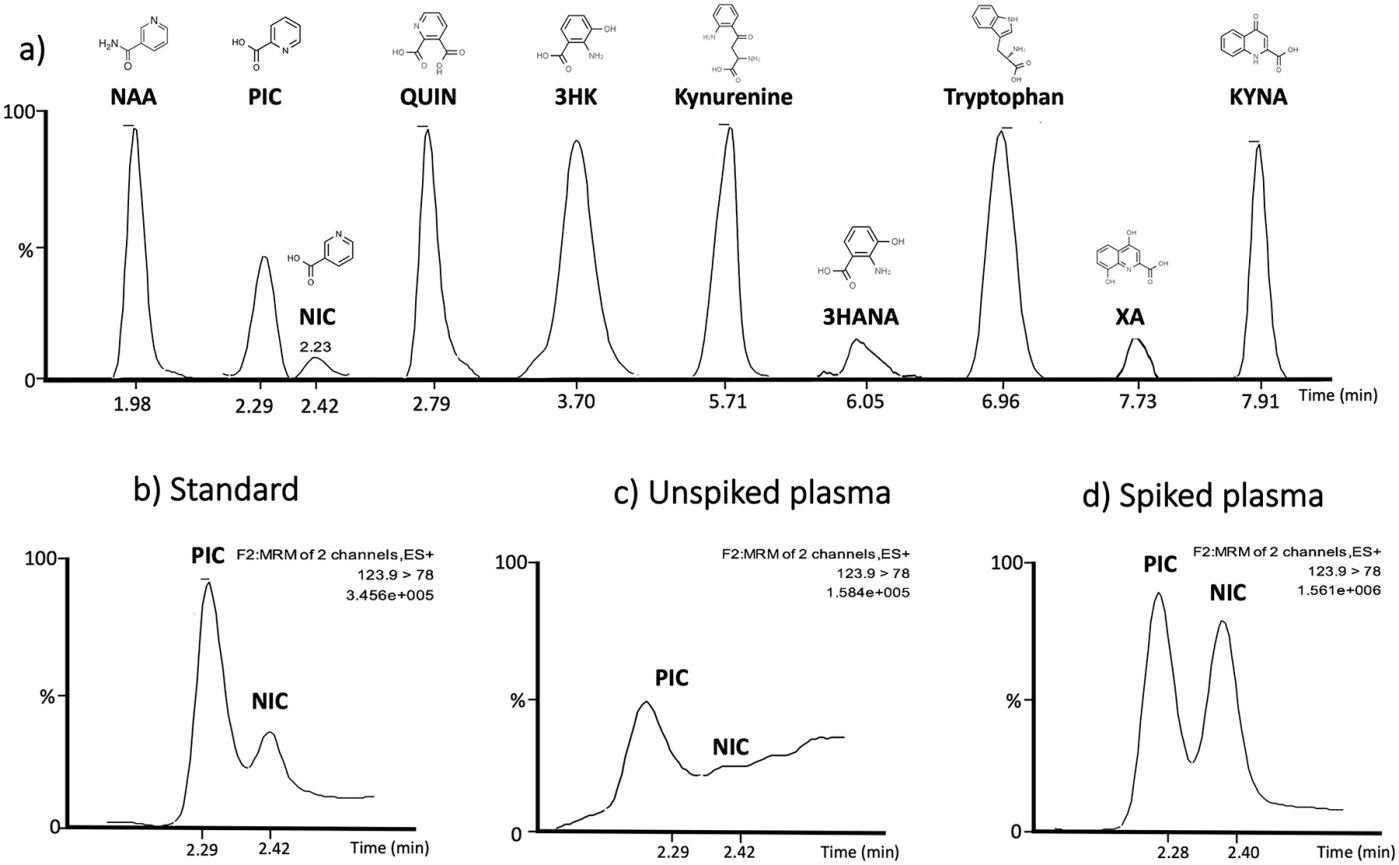
(a) Chromatograms from tryptophan and kynurenine metabolites in plasma showing respective retention times and chemical structures. Chromatograms illustrating the separation of the two isomers picolinic acid (PIC) and nicotinic acid (NIC), (b) in standard solution (6 nM), (c) in human plasma, and (d) in spiked plasma showing that the separation stays intact at higher concentration (1 μM). ES+, MRM and TIC is representing PIC. Abbreviations: ES+, Positive electrospray ionization mass spectrometry; MRM, Multiple reaction monitoring; TIC, Total ion current.

Chromatograms for all peaks are presented in Figure 2a and the specificity of the method for separation of the isomers PIC and NIC is presented in Figure 2b to d. The chromatograms clearly show that all peaks are free from any interference and that the method can clearly separate the 2 isomers, PIC and NIC in a standard solution (Figure 2b), plasma sample (Figure 2c) and in a plasma sample spiked with 1 μM of PIC and 1 μM NIC.

#### Assay precision, accuracy and matrix effect

Accuracy and precision were determined in spiked human plasma with 2 different concentrations covering the range of concentrations found in human plasma. Intra-assay accuracy (repeated measurements within the same experiment, *n* = 6) were stable for all metabolites (in both high and low concentrations) with a range between 87 to 110% of target (see Table 2). Intra-assay precision ranged between 0.9% to 4.4% relative standard deviation (RSD, Table 2). The inter-assay accuracy ranged between 85.2% to 114.8% and the inter-assay precision ranged between 1 and 14% RSD (Table 2). All metabolites fulfilled, in this regard, the criteria for US FDA and EMA guidelines accepted variation (less than 15%). 3-HANA was the only metabolite that had unsatisfactory variation in inter-assay accuracy with 79.2% of target tested (high concentration).

Endogenous concentrations of all analytes are present in human plasma and matrix effects were therefore determined with IS only and the matrix factor of IS calculated. Matrix effects for all the metabolites were moderate and are presented in Table 2.

#### Stability

The baseline concentration of the metabolites (mean ± SD, *n* = 6) were: tryptophan (34.9 µM ± 4), kynurenine (2.3 µM ± 0.3), KYNA (34.6 nM ± 13.3), 3-HK (27.9 nM ± 5.1), QUIN (162.7 nM ± 37), PIC (36.8 nM ± 14.3), NAA (178 nM ± 76.8), 3-HANA (43.7 nM ± 37.5), and XA (14.3 nM ± 7.6). The percent change of tryptophan, kynurenine, KYNA, 3-HK, QUIN, PIC and NAA were less than 4% and ranged between 96.7% and 103.6% in samples stored at room temperature for 30 minutes, 1, 2, 3, and 4 hours (see Table 3). 3-HANA and XA showed good stability at room temperature up to 2 hours (less than 15% variation) but 3-HANA had 19% variation after 4 hours of storage at room temperature and XA had 20% variation after 3 hours of storage at room temperature (see Table 3).

**Table 3.** Storage at room temperature expressed in % change.

Compound	Baseline mean ± SD (*n* = 6)	30 minutes (*n* = 6)	1 hour (*n* = 6)	2 hours (*n* = 6)	3 hours (*n* = 6)	4 hours (*n* = 6)
Tryptophan	34.9 µM ± 4	102.2 ± 2.9	102.3 ± 2.9	99.5 ± 2.5	100.5 ± 1.6	101.5 ± 1.9
Kynurenine	2.4 µM ± 0.3	102.4 ± 6.0	101.8 ± 4.8	99.6 ± 5.8	99.1 ± 1.7	101.1 ± 6.5
KYNA	34.6 nM ± 13.3	97.3 ± 7.1	97.1 ± 5.9	96.9 ± 7.0	96.7 ± 6.8	98.1 ± 6.1
3-HK	27.8 nM ± 5.1	100.7 ± 2.7	100 ± 4.6	97.5 ± 6.5	98.9 ± 3.2	102.3 ± 7.5
QUIN	161.7 nM ± 37	102.1 ± 5.1	103.1 ± 6.1	100 ± 3.8	100.8 ± 5.7	103.6 ± 6.1
PIC	36.8 nM ± 14.3	100.2 ± 5.2	99.2 ± 3.4	100.9 ± 3.0	97.4 ± 5.6	99.5 ± 5.8
NAA	178 nM ± 76.8	101.7 ± 3.8	101.5 ± 4.9	101.7 ± 3.4	100.5 ± 4.4	101.4 ± 5.1
3-HANA	43.7 nM ± 37.5	106 ± 10.3	104.7 ± 12.8	114.3 ± 12.7	109.7 ± 11.9	119.1 ± 13.2
XA	14.3 nM ± 7.6	95.1 ± 52.1	85.7 ± 40.1	88.9 ± 42.7	80.5 ± 38.2	96.4 ± 54.8

Compared with the baseline (the concentrations after the first thawing) considered 100%. For baseline and all freeze-thaw cycles, n = 6.

Abbreviations: KYNA, kynurenic acid; QUIN, quinolinic acid; PIC, picolinic acid; NAA, nicotinamide; 3HK, 3-hydroxykynurenine; XA, xanthurenic acid; 3HANA, 3-hydroxyanthranilic acid.

The stability of kynurenine metabolites before the very first freeze-thaw cycle and after 24 hours on the bench at room temperature was analyzed in 4 additional subjects. Mean baseline concentrations in fresh human plasma, analyzed within 30 minutes after blood drawing, were tryptophan (30.9 µM ± 5.3), kynurenine (2.2 µM ± 0.3), KYNA (44.3 nM ± 1.0), 3-HK (28.8 nM ± 7.6), QUIN (144.5 nM ± 18.8), PIC (80.3 nM ± 33.1), NAA (130.2 nM ± 91.8), 3-HANA (29.6 nM ± 6.5) and XA (20.9 nM ± 6).

The percent stability after 24 hours at room temperature ranged between 88.7% to 110.0% for tryptophan, kynurenine, KYNA, 3-HK, PIC, NAA and 3-HANA (see Table 4). QUIN and XA had 132.4% and 69.5% change respectively (see Table 4). All metabolites, with the exception of 3-HANA (126.4% variation), were stable and had less than 10% variation after the first freeze-thaw cycle. The percent increase in 3-HANA in all 4 subjects were found to be similar. More precisely, 3-HANA in all 4 subjects increased by 25.4, 24.7, 26.2, and 29.2%, respectively.

**Table 4.** Percentage change compared to baseline.

Compound	Baseline mean ± SD (*n* = 4)	Thawing 1 (*n* = 4)	24 hours on room temperature (*n* = 4)
Tryptophan	30.9 µM ± 5.3	102 ± 1.3	98.0 ± 4.7
Kynurenine	2.2 µM ± 0.3	105.9 ± 6.0	93.7 ± 4.4
KYNA	44.3 nM ± 10	101.6 ± 2.2	97.6 ± 5.5
QUIN	144.5 nM ± 18.8	110.2 ± 9.2	132.4 ± 5.1
PIC	80.3 nM ± 33.1	98.7 ± 1.6	95.2 ± 4.6
NAA	130.2 nM ± 91.8	107.7 ± 11.7	96.0 ± 0.04
3-HK	28.8 nM ± 7.6	99.9 ± 3.2	88.7 ± 1
3-HANA	29.6 nM ± 6.5	126.4 ± 2.0	111.7 ± 3.9
XA	20.9 nM ± 6	99.5 ± 2.7	69.5 ± 28.9

Compared with baseline, (the concentrations of kynurenines directly after blood drawing) (*n* = 4), considered 100%.

Abbreviations: KYNA, kynurenic acid; QUIN, quinolinic acid; PIC, picolinic acid; NAA, nicotinamide; 3HK, 3-hydroxykynurenine; XA, xanthurenic acid; 3HANA, 3-hydroxyanthranilic acid.

The percent stability after 2, 3, and 4 freeze-thaw cycles for tryptophan, kynurenine, KYNA, 3-HK, QUIN, PIC and NAA ranged between 95.3% and 103.2%. The only metabolite affected by repeated freeze-thaw cycles is XA that showed a 20% decrease after the fourth freeze-thaw cycle (see Table 5).

**Table 5.** Percent change after repeated freeze-thaw cycles.

Compound	Baseline mean ± SD (*n* = 6)	Thawing 2 (*n* = 6)	Thawing 3 (*n* = 6)	Thawing 4 (*n* = 6)
Tryptophan	34.9 µM ± 4	100.5 ± 1.9	99.9 ± 4.5	101 ± 1.4
Kynurenine	2.4 µM ± 0.3	101 ± 5.4	102.9 ± 6.5	100.1 ± 3.1
KYNA	34.6 nM ± 13.3	99.3 ± 2.4	99.7 ± 5.8	95.3 ± 7.9
3HK	27.9 nM ± 5.1	101.3 ± 3	103.2 ± 3	97.8 ± 2.5
QUIN	161.7 nM ± 37	100.5 ± 3.4	103.1 ± 9.8	98.4 ± 4.2
PIC	36.8 nM ± 14.3	100.1 ± 2.1	100 ± 3.5	101 ± 2
NAA	178 nM ± 76.8	101.8 ± 3.4	99.6 ± 4.4	102.5 ± 5.9
3-HANA	43.7 nM ± 37.5	103 ± 6.8	106.7 ± 13.3	104.3 ± 9.5
XA	14.3 nM ± 7.6	90.8 ± 49.6	86.7 ± 39.9	80.8 ± 40.2

Compared with the baseline (the concentrations after the first thawing) considered 100%. For baseline and all freeze-thaw cycles, *n* = 6.

Abbreviations: KYNA, kynurenic acid; QUIN, quinolinic acid; PIC, picolinic acid; NAA, nicotinamide; 3HK, 3-hydroxykynurenine; XA, xanthurenic acid; 3HANA, 3-hydroxyanthranilic acid.

### Effects on plasma kynurenine metabolite levels following one session of SIE

Tryptophan and 6 of its metabolites were successfully detectable in all plasma samples and therefore included in the statistical analyses. No differences were found in baseline levels of tryptophan, kynurenine, KYNA, 3HK, QUIN, PIC, NAA between young and old subjects (see Table 6).

**Table 6.** Baseline kynurenine metabolite levels in young and old subjects.

Compound	Young baseline	Old baseline	*P* values*
Tryptophan	43.8 µM ± 4	43.7 µM ± 4.3	.97
Kynurenine	2.5 µM ± 0.2	3.0 µM ± 0.3	.25
KYNA	49.5 nM ± 5.4	56.2 nM ± 15.1	.63
3HK	33.7 nM ± 4.4	43.6 nM ± 8.9	.35
QUIN	422.0 nM ± 61.4	404.8 nM ± 62.1	.84
PIC	58.4 nM ± 7.4	92.1 nM ± 29.1	>.99
NAA	127 nM ± 18.8	97.6 nM ± 9.6	.25

* Mann-Whitney *U*-test.

Abbreviations: KYNA, kynurenic acid; QUIN, quinolinic acid; PIC, picolinic acid; NAA, nicotinamide; 3HK, 3-hydroxykynurenine.

**Figure 3.**
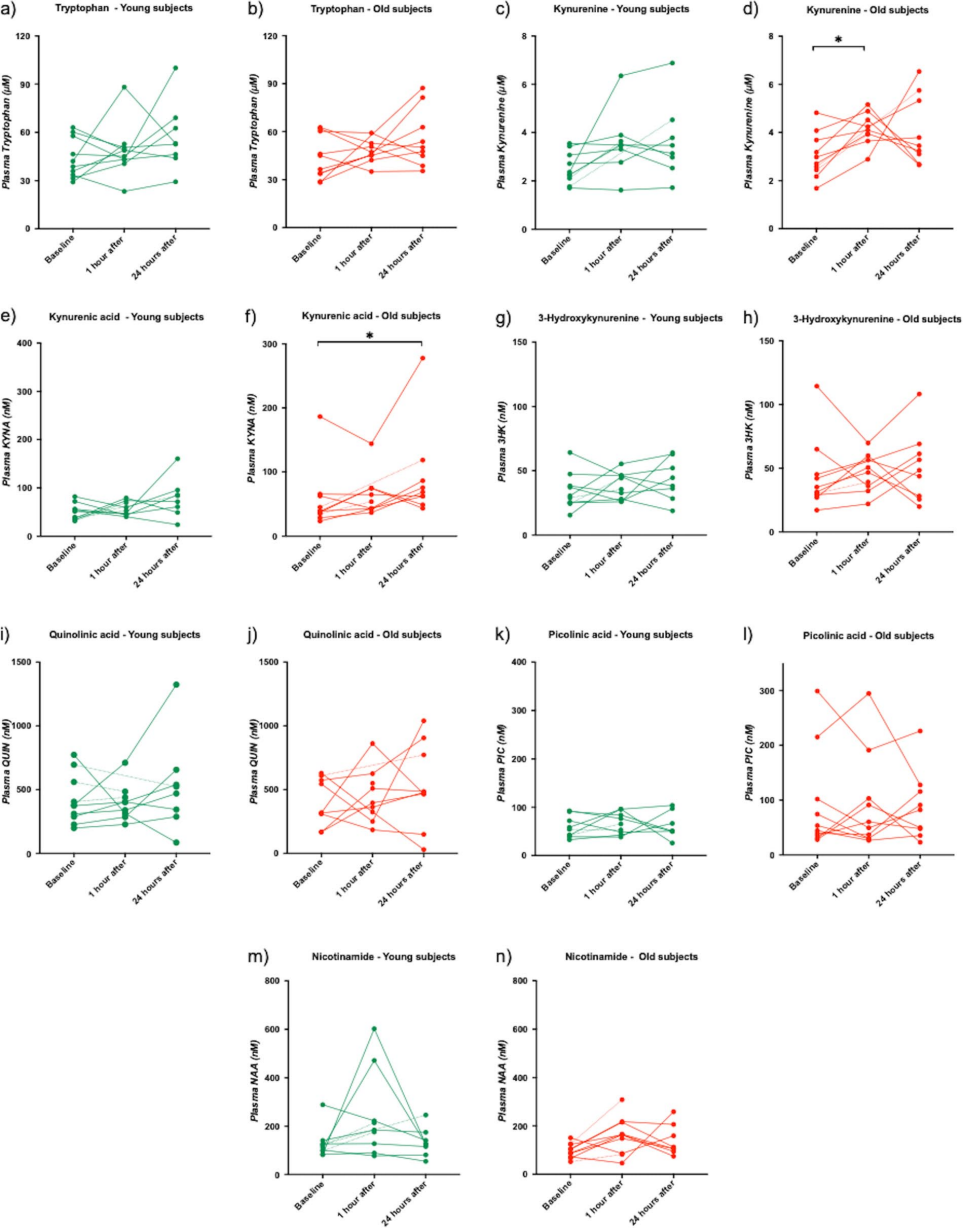
Plasma concentrations of kynurenine metabolites at baseline, 1 hour and 24 hours after SIE, in young (green) and old (red) subjects. Each dot represents the concentration of respective metabolite in nM (µM for tryptophan and kynurenine) of a single plasma sample. A dotted line is used when a timepoint data is missing for the subject: (a) tryptophan - young subjects, (b) tryptophan - old subjects, (c) kynurenine - young subjects, (d) kynurenine - old subjects, (e) kynurenic - young subjects, (f) kynurenic - old subjects, (g) 3-hydroxykynurenine - young subjects, (h) 3-hydroxykynurenine - old subjects, (i) quinolinic acid - young subjects, (j) quinolinic acid - old subjects, (k) picolinic acid - young subjects, (l) picolinic acid - old subjects, (m) nicotinamide - young subjects, and (n) nicotinamide - old subjects. *P < .05; **P < .01 versus baseline, Kruskal-Wallis post-hoc Dunn’s.

In young subjects, a tendency toward an increase in plasma kynurenine levels was observed 1 hour after a single session of SIE (*P* = .06), but plasma kynurenine was not altered 24 hours after SIE (Figure 3c). Plasma KYNA levels did not change after one (1) hour of SIE, but a tendency toward an increase in plasma KYNA levels was observed 24 hours after 1 session of SIE (*P* = .053) (Figure 3e). One session of SIE did not affect tryptophan (Figure 3a), 3HK (Figure 3g), QUIN (Figure 3i), PIC (Figure 3k), or NAA (Figure 3m) at any timepoint tested (1 hour and 24 hours after SIE).

In old subjects, plasma kynurenine levels were significantly increased one (1) hour after SIE (*P* = .03) (Figure 3d). After 24 hours, plasma KYNA levels were significantly increased (*P* = .02) (Figure 3d). No effect on plasma levels of tryptophan one (1) hour and 24 hours after SIE (Figure 3b), kynurenine 24 hours after SIE (Figure 3d), KYNA 1 hour after SIE (Figure 3f), 3HK one (1) hour and 24 hours after SIE (Figure 3h), QUIN 1 hour and 24 hours after SIE (Figure 3j), PIC one (1) hour and 24 hours after SIE (Figure 3l) and NAA one (1) hour and 24 hours after SIE (Figure 3n) was observed (One-way ANOVA, non-parametric test, Kruskal-Wallis).

## Discussion

In the present study, we have further developed a sensitive method for simultaneous quantification and detection of several kynurenine metabolites in human plasma. We have validated the method and used it to investigate the stability of kynurenine metabolites in plasma as well as for assessing if 1 session of SIE changes the concentration of these metabolites in young and old healthy adults.

The bioanalytical method showed good linearity, precision and accuracy for all kynurenine metabolites: tryptophan, kynurenine, QUIN, KYNA, PIC, NAA, NIC, XA, 3-HK and 3-HANA. The 3-HANA was the only metabolite that had a slight deviation from an accepted % change of accuracy in samples spiked with a high concentration (400 nM). However, the accuracy for 3-HANA spiked with the lower concentration (40 nM, representing the values found in human plasma) was acceptable. The selectivity of the method for measuring plasma levels of all metabolites was good and the LLOQs (S/N > 10) clearly cover the endogenous plasma levels for all metabolites with an exception for NIC. Furthermore, the present LC-MS/MS protocol clearly defines the isomers PIC and NIC as 2 separate peaks.

One parameter of importance when analyzing human metabolites is stability, as storage or processing conditions may result in degradation before analysis. For this reason, FDA Guidance includes a section on sample stability and, in accordance with those guidelines, all metabolites were tested with regard to the impact of storage at room temperature (for 30 minutes, 1 hour, 2 hours, 3 hours, 4 hours, and 24 hours) and following 1-4 freeze-thaw cycles. In line with what we have previously shown for central kynurenine metabolite concentrations, plasma tryptophan, kynurenine, KYNA, PIC, NAA, and 3-HK were also found to be stable when left at room temperature for up to 4 hours. However, our results suggest that for analysis of XA and 3-HANA, plasma should not be kept at room temperature longer than 2-3 hours, since that could compromise the results, giving false increased signal. The stability test also showed that storage at room temperature for a long time (24 hours) could affect plasma concentrations of QUIN. Furthermore, all metabolites except 3-HANA, show stability over 4 repeated freeze-thaw cycles. Since the increase in plasma 3-HANA was similar in 4 subjects, we suggest that this metabolite could be included in future analysis however, further evaluation of its stability might be needed.

The next aim of the current study was to apply the new method of analysis on a cohort of untrained young and old healthy subjects performing 1 single SIE. Thus, plasma kynurenine metabolites were analyzed before and after 1 session of SIE in recreationally active individuals. In old adults, we found an increase in plasma kynurenine levels 1 hour after, and an increase in plasma KYNA levels 24 hours after 1 SIE session. In young subjects, tendencies toward increased plasma kynurenine levels after 1 hour and in plasma KYNA levels after 24 hours were observed but did not reach the significance threshold.

Here, the resistance in the training protocol was related to weight, where muscle mass is known to be reduced with age, suggesting that the relative training intensity was higher in old subjects^31^ which could account for the significant results in this cohort compared to the trends observed in younger subjects. However, the difference between young and old subjects may also relate to age-dependent differences in immune activity,^32,33^ or skeletal muscle physiology, in turn explaining an enhanced production capacity of kynurenines in elderly people.^34,35^ To this end, it would be interesting, in the future, to measure levels of inflammation in the subjects to see whether baseline inflammation might affect the profile of kynurenines generated in response to exercise.

Previous studies have shown that resistance or endurance-type physical activity induces tryptophan breakdown in healthy adults, resulting in decreased tryptophan^22,23,36,37^ and increased kynurenine levels^37^ while other studies show no change,^23,38-40^ or even the opposite effect, with tryptophan levels increased and kynurenine levels decreased.^36,41^ There is also 1 study in patients with multiple sclerosis (MS) showing decreased tryptophan concentration in plasma of relapsing remitting patients with MS following acute endurance exercise and also after 3 weeks of chronic endurance exercise.^42^ The effects of physical exercise on plasma KYNA levels have also been analyzed in several studies. Thus, KYNA, in both CSF and plasma is shown to be increased in healthy subjects performing endurance exercise.^22,23,36,43,44^ On the other hand, resistance and non-endurance types of exercise do not seem to influence plasma KYNA levels.^23,40,43^ One study showed an acute effect of high intensity exercise in healthy subjects, resulting in increased KYNA directly after exercise and then back to baseline after 1 hour.^45^ Endurance training also increases plasma QUIN levels,^22,23,43^ while resistance exercise does not.^23,43^ It is clear from these works that the type of exercise (endurance, resistance or non-endurance) and baseline fitness levels (eg, someone running a marathon^22^ or cycling long-distances^43^ is well-trained to perform that task) differentially influence metabolism along the kynurenine pathway. Interestingly, 1 study has shown that tryptophan and kynurenine levels correlate positively with aerobic fitness capacity (VO_2 max_)^37^ supporting subject fitness as a parameter to consider. It should thus be kept in mind that our results show age-dependent effects of 1 SIE session in recreationally active but essentially untrained individuals and provides information only on how the kynurenine pathway might be affected in the early-phases of training.

The differences in levels of kynurenine metabolites seen in the above studies may also be due, in part, to the time of sampling following not only termination but also initiation of the exercise. For example, transient changes in plasma kynurenine metabolites were observed by Schlittler et al^43^ where KYNA concentration peaked at 1h and was back to baseline at 24 hours following a bout of endurance exercise consisting of a 150-km road cycling time trial. However, this 1h sample actually represents, roughly and on average, 13 hours of elapsed time from exercise initiation to sampling (and roughly 37 hours in the case of the 24 hours sample). In contrast, our SIE session consisted of an 8 minutes warm-up followed by 6 repeated all-out bouts of cycling (30 seconds)/rest (4 minutes) for 27 minutes (35 minutes total) for an elapsed time of approximately 1.5 hours from the start of exercise to the first sampling point (1 hour post-exercise termination) where we did not observed elevations in KYNA. Thus, although both studies use a sampling point of 1h after the end of the exercise, the length of time in which muscular and metabolic activity along with metabolite flux through the kynurenine pathway was sustained actually differs. Such divergence is likely presence across many studies, and, as mentioned above, exercise-type and subject fitness-level likely influence these specific results as well. Training intensity is yet another factor to consider when measuring kynurenine metabolites and comparing across studies. For instance, in healthy subjects, exercise intensity affects the kynurenine metabolites differently, by increasing KYNA plasma levels after hypertrophic strength loading, but not after maximal strength loading.^45^ Irrespective of lab-to-lab variations in methods and design, together, these sometimes-contradictory studies suggest that plasma kynurenine metabolites can change within temporally limited windows and that the direction of change depends on the type, length and intensity of the exercise performed. The current investigation also presents some limitations with respect to the experimental design such as lack of physiological parameters controlling for “all-out” performance and uncontrolled food intake before plasma sampling. Controlled food intake is important since tryptophan in food will change plasma levels of tryptophan and kynurenine metabolites.^46^ However, it should be noted that in the field of training studies investigating tryptophan and kynurenine pathway metabolites, very few have controlled for food intake during the experiment and this could also contribute to the variability observed across studies.

The results of the present study suggest that 1 session of SIE influences kynurenine and KYNA in old people but not in the young. It is possible that increased kynurenine metabolite concentrations could be triggered, in part, by the inflammatory stimulus due to high intensity exercise and therefore might necessitate repeated sessions to manifest itself in the young. Future studies are required to reveal if several bouts of SIE over a longer period are able to influence tryptophan and kynurenine metabolite concentration in young subjects.

In conclusion, the present paper provides a detailed protocol for an accurate, robust and precise bioanalytical method for the simultaneous quantification of 10 plasma kynurenine metabolites. In addition, we show that 1 session of SIE influences kynurenine and KYNA in old people but not in the young, however future studies should examine whether repeated bouts of SIE are able to affect tryptophan and kynurenine metabolite concentration in young subjects, given the recent appeal of short-duration exercise and the link between aerobic exercise and decreased depressive symptoms. More studies are also needed to understand why different exercise-induced effects on the kynurenine system are recorded in young versus old healthy subjects.
